# A computational analysis of the three isoforms of glutamate dehydrogenase reveals structural features of the isoform EC 1.4.1.4 supporting a key role in ammonium assimilation by plants

**DOI:** 10.1186/1745-6150-1-38

**Published:** 2006-12-15

**Authors:** Emmanuel Jaspard

**Affiliations:** 1 UMR 1191 Physiologie Moléculaire des Semences, Université d'Angers – INRA – INH, Angers, France

## Abstract

**Background:**

There are three isoforms of glutamate dehydrogenase. The isoform EC 1.4.1.4 (GDH4) catalyses glutamate synthesis from 2-oxoglutarate and ammonium, using NAD(P)H. Ammonium assimilation is critical for plant growth. Although GDH4 from animals and prokaryotes are well characterized, there are few data concerning plant GDH4, even from those whose genomes are well annotated.

**Results:**

A large set of the three GDH isoforms was built resulting in 116 non-redundant full polypeptide sequences. A computational analysis was made to gain more information concerning the structure – function relationship of GDH4 from plants (*Eukaryota*, *Viridiplantae*). The tested plant GDH4 sequences were the two ones known to date, those of *Chlorella sorokiniana*. This analysis revealed several structural features specific of plant GDH4: (i) the lack of a structure called "antenna"; (ii) the NAD(P)-binding motif GAGNVA; and (iii) a second putative coenzyme-binding motif GVLTGKG together with four residues involved in the binding of the reduced form of NADP.

**Conclusion:**

A number of structural features specific of plant GDH4 have been found. The results reinforce the probable key role of GDH4 in ammonium assimilation by plants.

**Reviewers:**

This article was reviewed by Tina Bakolitsa (nominated by Eugene Koonin), Martin Jambon (nominated by Laura Landweber), Sandor Pangor and Franck Eisenhaber.

## Open peer review

Reviewed by Tina Bakolitsa (nominated by Eugene Koonin), Martin Jambon (nominated by Laura Landweber), Sandor Pangor and Franck Eisenhaber. For the full reviews, please go to the Reviewers' comments section.

## Background

There are three isoforms of GDH. According to the following reaction:

2-oxoglutarate + NH_4 _^+ ^+ NAD(P)H + H^+ ^⇔ glutamate + H_2_O + NAD(P)^+ ^GDH EC 1.4.1.2 (GDH2) catalyses essentially the formation of 2-oxoglutarate using NAD(P)^+ ^[1]; GDH EC 1.4.1.3 (GDH3) catalyses both the formation of 2-oxoglutarate and the reverse reaction, thus exhibiting a dual coenzyme specificity [NAD(P)^+^/NAD(P)H] [2]; GDH EC 1.4.1.4 (GDH4) catalyses the formation of glutamate using NAD(P)H [3,4]. For example, GDH4 is known to play an anabolic role in ammonium assimilation in the yeast *Candida utilis *[5]. Nevertheless, it is considered that the major route of ammonium assimilation in plants involves the glutamine synthetase – glutamate synthase couple [6]. However, high ammonium concentration deactivates glutamine synthetase and induces GDH [7,8]. In fact, data on the actual role of GDH4 from plants either in ammonium assimilation or in the formation of 2-oxoglutarate are controversial.

Several three-dimensional structures of GDH from prokaryotic and eukaryotic organisms have been resolved [9-11]. All GDHs described to date are homo-oligomeric proteins and the most striking differences between the three isoforms arise from the primary and the quaternary structures. GDHs were classified into four families on the basis of the length of the polypeptide chain and the number of subunits [12]. GDH2 are dimeric (unique case for this enzyme) [13], tetrameric [14] or hexameric [15]; GDH3 are essentially hexameric [16]; GDH4 are tetrameric [17] or hexameric [18].

A bioinformatics analysis of a large set of the three isoforms of GDH was made to gain more information concerning the structure – function relationship of GDH4 from plants (*Eukaryota*, *Viridiplantae*). The tested plant GDH4 sequences were the two ones known to date, those of *Chlorella sorokiniana *[19].

The following characteristics were found specific to GDH4 from *Chlorella sorokiniana*: (i) a small N-terminal region and no C-terminal extension; (ii) a central domain with the substrates and the nucleotide-binding sites but without a structure called antenna ; (iii) a second putative coenzyme-binding motif whose fingerprint sequence is GVLTGKG ; (iv) four residues (Lys, Ser, Arg and Thr) involved in the binding of the reduced form of the coenzyme, NADPH. A model of the structure of the active site of GDH4 from *Chlorella sorokiniana*, with NADPH and glutamate, is proposed. The role of these two coenzyme-binding motifs and of these four residues in the stabilization of the reduced form of NADP is discussed to explain the functional specificity of plant GDH4 in the formation of glutamate.

## Results

### Organization of the GDH subunits

**Figure 1:**
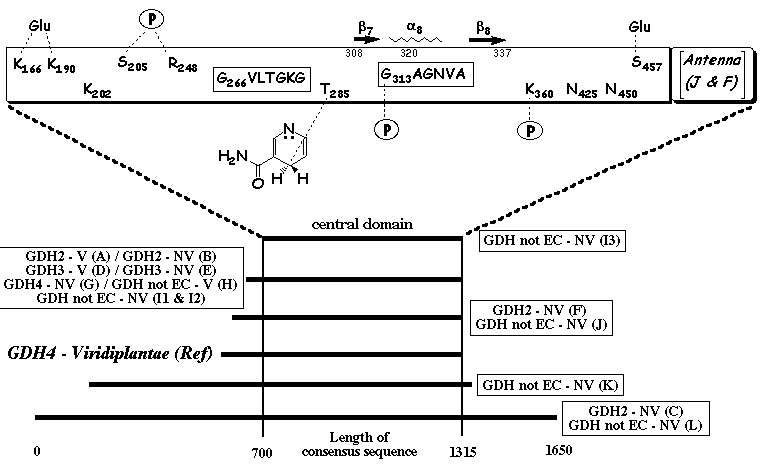
**Global organization of GDH subunits**. All GDHs share a common pattern called the central domain flanked by an N-terminal region of various lengths and, for large GDHs, by a shorter C-terminal one. The upper scheme shows the structural features specific of the central domain from plant GDH4. The length indicated includes the gaps generated by the alignment.

For each subset (Table 1), sequences of the complete GDH subunits were aligned and the best full consensus sequence was determined by testing various combinations of matrix and gap penalty parameters. The 15 full consensus sequences were themselves aligned using the same parameters as for the determination of each full consensus sequence. The result (Fig. 1) shows that GDH subunits contain one, two or three regions. The smallest GDH (subset I3) contain only the pattern common to all GDHs, called the central domain. All other GDH subunits contain either only an N-terminal region of various lengths, or contain an N-terminal and a C-terminal region (GDHs from subsets C, K and L). Such an organization of the GDH subunits is in agreement with previous descriptions [12,20].

**Table 1:** Number of polypeptide sequences of GDH for each subset (letter)

EC number	*Viridiplantae*				*not Viridiplantae*			
	
	L1	L2	L3	L4	L1	L2	L3	L4
1.4.1.2	9 (A)				7 (B)			1 (C)
1.4.1.3	1 (D)				6 (E)	7 (F)		
1.4.1.4		2 (*Ref*)			15 (G)			
not					13 (I1)			
EC	6 (H)				18 (I2)	5 (J)	5 (K)	7 (L)
classified					14 (I3)			

Length range of polypeptide chains (number of amino acids): L1 = [411 : 470] – L2 = [503 : 558] – L3 = [1029 : 1106] – L4 = [1607 : 1651]

### Comparison of the central domain of GDHs

The alignment of full consensus sequences was helpful to extract from each subset the consensus sub-sequence corresponding to the central domain of GDHs. Indeed, the full consensus sequence of subset I3 was used as the template since it has neither apparent N- nor C-terminal region. The 15 consensus sub-sequences were themselves aligned. The three-dimensional structure of various GDHs reveals that the coenzyme is bound in an extended conformation with the nicotinamide moiety deep in the cleft between the substrate and the coenzyme domains. In the case of GDH4 from *Chlorella sorokiniana *(Fig. 1), the nicotinamide ring is probably adjacent to a very well conserved motif containing three lysine residues: *K*^166^-G-G-x-R-x(12,23)-L-x(6)-*K*^190^-x(4,6)-P-x-G-G-x-*K*^202 ^(according to amino acids position of *Ref*). Lys^190 ^and Lys^202 ^are found in all subsets. For large GDHs (subsets C, K and L), there is a conservative substitution of Lys^166 ^into Arg and, moreover, there is an insertion of nine amino acids in the case of subset K. This Lys-rich region has been assigned as the [2-oxoglutarate/Glu]-binding site [21]. Lys^166 ^and Lys^190 ^establish salt bridges with the two carboxyl groups of 2-oxoglutarate or Glu. Lys^202 ^is involved in catalysis rather than in the binding of the [substrate/product] [22].

**Figure 2:**
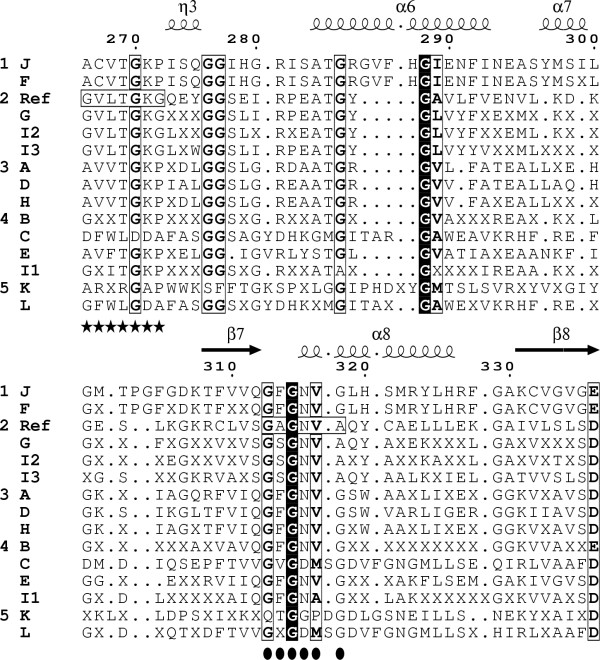
**The two dinucleotide-binding motifs of plant GDH4**. The part of each consensus sequence corresponding to the central domain of GDHs was extracted using the full consensus sequence of subset I3 as the template. The 15 subsets (J to L) are presented in five groups (1 to 5) according to the percentages of identity. Secondary structures indicated above the alignment were generated using the bovine GDH3 complexed with NADPH and glutamate (PDB # 1HWZ) as the template. Amino acid position indicated above the alignments is that of plant GDH4 (*Ref*). Plain vertical boxes. amino acids identical for all consensus sub-sequences. Open vertical boxes. amino acids whose homology between all consensus sub-sequences was greater than 60%. The letter "X" accounts for an amino acid whose identity level was less than 60% after the first alignment of full consensus sequences. The found dinucleotide-binding motif G^266^VLTGKG^272 ^(*Ref*) and the dinucleotide-binding motif G^313^AGNVA^318 ^(*Ref*) included in the N-terminal part of the Rossmann fold (β7-α8-β8) are indicated at the bottom of the frame with asterisks and circles, respectively.

Secondary structures were built using as the template the bovine GDH3 complexed with NADPH and glutamate (PDB # 1HWZ). A βαβ fold is found in the coenzyme-binding sub-domain (β7-α8-β8, Fig. 2). This Rossmann fold begins with the motif G^313^AGNVA^318 ^in the case of plant GDH4 (*Ref*). By comparison, the motifs described in the literature are GXGXXG, GXGXXA and even GXGXXS as for example in the case of very large GDH from *Streptomyces clavuligerus *[12]. However, the alignment indicates that the actual motifs are more complex and that, among the short GDHs, the main differences arise from the nature of the residues in the second and the last position of the motifs. Such a higher complexity of the signature for the dinucleotide-binding motif makes it possible to discriminate more precisely between the three isoforms: (i) the hexapeptide GAGNVA is found for GDH4 from *Viridiplantae*; (ii) the hexapeptide GSGNVA is the signature for GDH4 from *not Viridiplantae *(subset G). The finding of the same motif for subsets G, I2 and I3, together with high percentage of identity between them (86% between G and I2, 70% between G and I3), suggest that GDHs not EC classified from subsets I2 and I3 are also GDH4; (iii) the hexapeptides GFGNVG or GFGNAG are found for subsets A, D, H (*Viridiplantae*) and for subsets B, E, I1 (*not Viridiplantae*); (iv) a very different heptapeptide G(Q) [V/T] [D/G] [M/P] [S/D]G, sharing the first and the third conserved Gly, is found for large GDHsfrom subsets C, K and L. The K_m _^NADPH ^values for the wild-type GDH4 from *Salmonella typhimurium *and for the mutant GDH K^286^E are 9.8 μM and 280 μM, respectively [23]. This indicates that the side chain of the equivalent Lys residue for plant GDH4 (Lys^360 ^of *Ref*, Fig. 1) might stabilize the reduced form of NADP by neutralising the negative charge of the 2' ribose phosphoryl group.

The central domains of GDHs from subsets F and J (GDHs from *not Viridiplantae *of length L2) contain 494 amino acids while that of *Ref *contains only 431 residues. 48 of these additive residues (horizontal box for subsets J and F, Fig. 1) form a helix – random coil structure called the antenna in the coenzyme-binding domain [24]. This antenna is involved in subunit interactions and allosteric regulation of the enzymatic activity of GDH. Moreover, the mutation of the Arg residue (sequences Q- [D,X]-*R*-I- [D,X]-G of subsets J and F) into Ser, decreases tenfold the activity of human GDH [25]. It has been shown that plants GDH do not possess this antenna [26] and indeed the GDH4 sequences of *Ref *do not contain this amino acid sequence. Therefore, the lack of the antenna motif and of this Arg residue is specific to plant GDH4. Finally, since subset F corresponds to GDH3, one can make the assumption that GDHs from subset J are also GDH3.

### Modeling of the active center of plant GDH4

**Figure 3:**
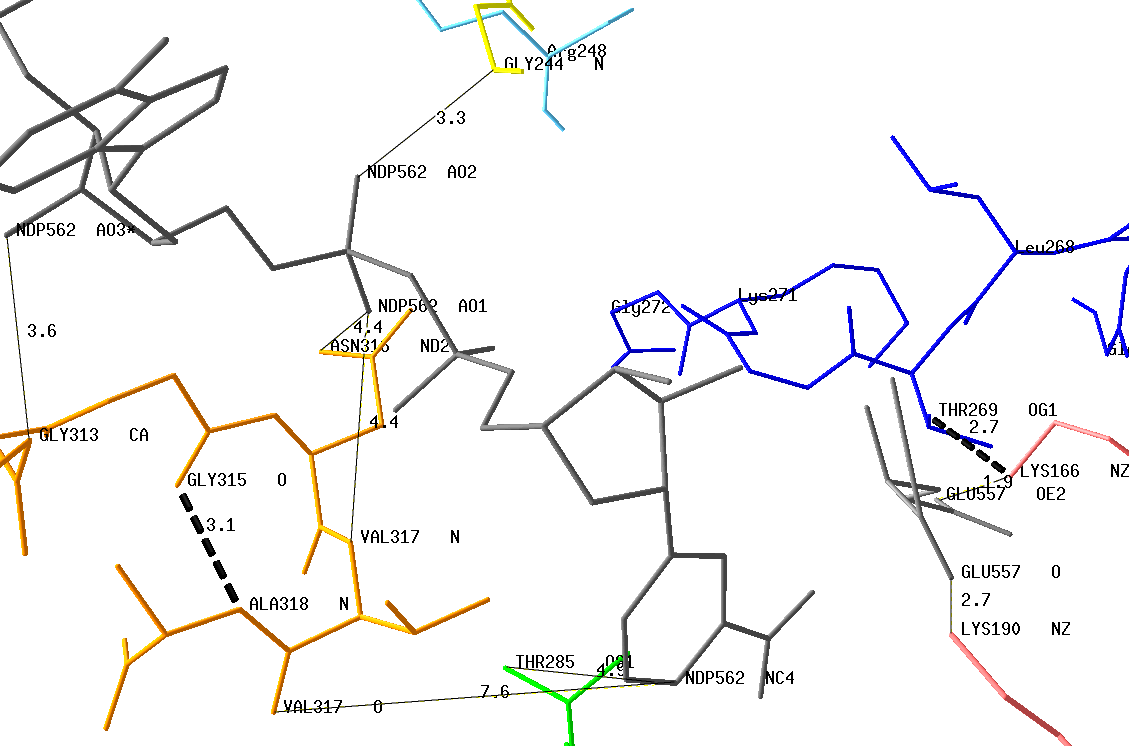
**Modeling of the coenzyme-binding motifs and key residues of plant GDH4 with NADPH (NDP^562^) and Glu**. Modeling of the coenzyme-binding motifs and key residues of GDH4 from *Chlorella sorokiniana *with NADPH (NDP^562^) and glutamate. Some interactions (plain lines) between the motif G^313^AGNVA^318 ^or key residues and the coenzyme are indicated. NDP^562^AO3 – Gly^313^CA; NDP^562^AO1 – Asn^316^ND2; NDP^562^AO1 – Val^317^N; NDP^562^AO2 – Gly^244^N and NDP^562^NC4 – Thr^285^OG1. The distances between the protonated carbon atom of the nicotinamide moiety (NDP^562^NC4) are too long for direct interactions with the motif G^313^AGNVA^318^. However, this motif is stabilized by the H-bond Gly^315^O – Ala^318^N (dotted line). Two distances (Glu^557^OE2 – Lys^166^NZ and Glu^557^O – Lys^190^NZ) are compatible with salt-bridges between the enzyme and glutamate. The position of the motif G^266^VLTGKG^272 ^is shown with the potential H-bond Lys^166^NZ – Thr^269^OG1. The figure was made with the program DeepView using a PDB-like file of GDH4 of *Chlorella sorokiniana *generated with the structure of bovine GDH3 (PDB # 1HWZ) as the template.

A theoretical 3D structure of GDH4 from *Chlorella sorokiniana *was calculated with the homology-modeling program ESyPred3D that creates a PDB-like file, using as the template the structure of bovine GDH3 (PDB # 1HWZ). This structure was chosen as the template for three reasons: (i) its length (501 amino acids) is similar to that of the GDH4 sequences of *Chlorella sorokiniana *(523 amino acids); (ii) considering the dual coenzyme specificity [NAD(P)^+^/NAD(P)H] of GDH3, it is assumed that its structure is closer to that of plant GDH4 than to any GDH2 structure; and (iii) the modeled data were obtained for the enzyme complexed to the reduced form of the coenzyme, NADPH. Using these two PDB files (the created PDB-like file for GDH4 from *Chlorella sorokiniana *and PDB # 1HWZ), a putative structure of the active center of GDH4 from *Chlorella sorokiniana *was modeled (Fig. 3) with the protein structure homology-modeling program DeepView.

The model reveals three interactions (NDP^562^AO3 – Gly^313^CA, NDP^562^AO1 – Asn^316^ND2 and NDP^562^AO1 – Val^317^N) between the coenzyme-binding motif G^313^AGNVA^318 ^and NADPH (NDP^562^). Moreover, this motif is stabilized by both the internal H-bond (Gly^315^O – Ala^318^N) and the interaction NDP^562^AO2 – Gly^244^N. This latter interaction was found by comparison with the H-bond network of the template bovine GDH3 (the equivalent of Gly^244 ^being Ser^170 ^in the case of bovine GDH3). As previously mentioned, Lys^166 ^and Lys^190 ^are known to be the [2-oxoglutarate/Glu]-binding sites [21]. The model shows two interactions (Glu^557^OE2 – Lys^166^NZ and Glu^557^O – Lys^190^NZ) whose distances are compatible with salt bridges with the two carboxyl groups of 2-oxoglutarate, *i.e*., the substrate of the reaction catalysed by plant GDH4.

### Evidence for a second reduced coenzyme-binding site in plant GDH4

Among the dehydrogenase family, aldehyde DH from the bacterium *Vibrio harveyi *is one of the most NADP-specific (K_m _^NADP ^is 150-fold lower than K_m _^NAD^) [27]. The sequence of aldehyde DH from *Vibrio harveyi *(GenBank # Q56694) and those of GDH4 from *Chlorella sorokiniana *are roughly of the same length (510 and 523 amino acids, respectively) with 28% identity and 52% similarity. Moreover, aldehyde DH from *Vibrio harveyi *is an oligomer of 50–55 kDa subunits such as GDH4 of *Ref*. Finally the nucleotide-binding motif for aldehyde DH of *Vibrio harveyi *is G^229^SVGGG^234 ^and is included in a Rossmann fold [28]. Such close functional and structural characteristics led me to compare these two enzymes using aldehyde DH from *Vibrio harveyi *(PDB # 1EZ0) as the template. The results are presented in the Figure 1: (i) three putative key residues for the binding of NADPH are localized in GDH4 from *Chlorella sorokiniana*: Lys^202^, Ser^205 ^and Arg^248^; (ii) the motif G^229^SVGGG^234 ^(aldehyde DH) is aligned with the motif G^266^VLTGKG^272 ^of GDH4 of *Ref *indicating that the latter is likely to be a second reduced coenzyme-binding motif characterizing plant GDH4. A model of this motif with NADPH and Glu is proposed (Fig. 3).

## Discussion

The functional specificity of GDH4 is the formation of glutamate using NADPH. The results suggest that the fingerprint sequence G^313^AGNVA^318 ^is the signature of one of the two coenzyme-binding motifs of plant GDH4. None of the distances calculated between the residues of this motif and the protonated carbon atom of the nicotinamide moiety (NDP^562^NC4) are compatible with interactions susceptible to stabilize the reduced form of this atom (Fig. 3). Nevertheless, this result is not so surprising. First, Gly^315 ^seems involved in maintaining the conformation of the motif (through an H-bond with Ala^318^N) rather than in the coenzyme specificity, since it is conserved in the three isoforms of GDH (Fig. 2). Second, three residues of this motif interact with other parts of NADPH: NDP^562^AO3 – Gly^313^CA, NDP^562^AO1 – Asn^316^ND2 and NDP^562^AO1 – Val^317^N. The second interaction underlines the difference in orientation of the coenzyme in the active site between GDH3 and GDH4 because the equivalent Asn residue of bovine GDH3 (Asn^254^) is H-bonded to the carboxyamide group of the nicotinamide ring [21]. Third, the mechanism of interaction between the adenine ribose and the fingerprint sequence GXGXX*G*/*A *depends (at least for NAD-dehydrogenases) on the nature of the residue occupying the last position of this motif but is independent of the coenzyme specificity [29].

Smith and coll. [30] have resolved the structure of three abortive complexes of bovine GDH3 (GDH-NADH-Glu-GTP, GDH-NADPH-Glu-GTP and GDH-NAD-2-oxoglutarate) and they have shown that NADH and NADPH bind to a second coenzyme site. The dissociation constants from these two sites are 57 and 700 μM, respectively [31].

There is evidence for the existence of a second coenzyme-binding site in plant GDH4 whose sequence is G^266^VLTGKG^272 ^(*Ref*; Fig. 1): (i) the perfect alignment of this motif with the motif GSVGGG of aldehyde DH; and (ii) the existence of three residues of GDH4 of *Ref *equivalent to residues of aldehyde DH known to play a key role in the binding of the coenzyme [28]: by analogy, Lys^202 ^(*Ref*) probably interacts with the 3'-hydroxy group of the coenzyme while Ser^205 ^and Arg^248 ^make an H-bond with the 2'-phosphate group. The modeling of the motif G^266^VLTGKG^272 ^with the coenzyme (Fig. 3) does not allow me to visualize these interactions but shows that its orientation towards the nucleotide could be stabilized through an H-bond between Lys^166^NZ and Thr^269^OG1. Moreover, a fourth residue (Thr^285^) probably makes an H-bond with the protonated carbon atom of the nicotinamide moiety (NDP^562^NC4).

Are the motif GVLTGKG and these four residues specific to plant GDH4 ? All subsets contain a Lys residue equivalent to Lys^202 ^of GDH4 of *Ref *and, except for large GDH (subsets C, K and L), they contain a Thr residue equivalent to Thr^285 ^and the short sequence equivalent to T^269^GK. However, only GDHs from subsets *Ref*, J, F, G, I2 and I3 contain an Arg residue equivalent to Arg^248^. Among them, only GDHs from subsets *Ref*, G, I2 and I3 contain a Ser residue equivalent to Ser^205 ^(followed in addition by the same positively charged sequence D^206^FD^208^). Finally, only GDHs from these four subsets contain exactly the motif G^266^VLTGKG^272^. Since subsets *Ref *and G correspond to GDH4, the motif G^266^VLTGKG^272 ^and the four residues (Lys^202^, Ser^205^, Arg^248 ^and Thr^285^) are likely to be structural features specific of plant GDH4.

A problem to estimate the actual activity of GDH4 arises from the co-existence of the two or even the three isoforms of GDH in a cell. In crude extracts containing the three isoforms, it is very difficult to measure specifically the reaction rate of GDH4, *i.e*., the rate of ammonium assimilation into glutamate. Instead, the overall rate of the three reactions is measured, masking or minimizing the actual velocity of GDH4. This under-estimation leads, most of the time, to the conclusion that this isoform is not active and, therefore, plays no role in ammonium assimilation. Moreover, there is sometimes confusion in the literature between the reaction catalysed and the isoform, the reaction of ammonium assimilation being associated to GDH2 instead of GDH4 [32]. When authors conclude that GDH is less active, it means that GDH2 is less active, not GDH4.

Despite a consensually accepted role of the glutamine synthetase – glutamate synthase couple in ammonium assimilation by plants [33-35], some data are in favor of such a role also for GDH4. For example, the expression of GDH from *Chlorella sorokiniana *in tobacco plants increased the growth rate and chlorophyll content, suggesting a better uptake and utilization of ammonium in plants [36]. Moreover, the production and the activity of GDH are induced when tobacco plants are grown on ammonium as the sole nitrogen source and the results indicate a dual role of GDH in the mitochondria at low ammonium concentration or in the cytosol at high ammonium concentration [37]. Such responses is likely the result of negative and/or positive homotropic effects between the two NAD(P)H binding-sites, allowing allosteric regulation of the enzymatic activity of GDH4 in the absence of antenna.

## Conclusion

The present study identifies a new coenzyme binding site in GDH4 with a potential regulatory role in the activity of this isoform. The existence of such a site allows for the design of genetic engineering experiments aimed at improving the efficiency of absorption and transportation of nitrogen. Other possible applications include enhancing the resistance of plants to environmental stresses (e.g., dehydration, elevated CO_2 _levels, hypoxia ...). Further experiments will be required to address these issues.

***Note added in proof****: *Since this work has been submitted, some GDH were re-annotated in databases. *Bos taurus *GDH # AAN15276 from subset J, initially classified as "non EC-classified", is actually GDH3. *Plasmodium falciparum *GDH # NP_702052 from subset I2 and *Helicobacter pylori *GDH # D64567 from subset I3, are now classified as GDH4. These new classifications confirm some conclusions made in the present paper.

## Methods

The search for the complete amino acids sequences of GDH was made at NCBI using the «Entrez» service. The sequences of the three GDH isoforms (EC 1.4.1.2, EC 1.4.1.3, EC 1.4.1.4) plus not «EC-classified» GDHs were selected by examination of the "*GenPept*" files. Only fully-annotated files were chosen and files mentioning «hypothetical», «like» «putative», «similar», «probable», «related», «partial» were discarded. The non-redundant sequences were retrieved by alignment using Multalin [38]. Finally, 116 non-redundant complete GDH sequences were obtained from 83 organisms representing the three domains, *Archaea*, *Bacteria *and *Eukaryota *(See additional file 1: Appendix1.pdf for the original data used to perform this analysis). These sequences were classified into 15 different subsets using the following criteria: the EC number, the length of the polypeptide chains and their belonging to *Viridiplantae *or not (Table 1). The tested subset called *Ref *contains the two sequences of GDH4 of *Chlorella sorokiniana *(*Viridiplantae*, *Chlorophyta*, GenBank # CAA41635 &CAA41636).

The sequences of each subset were aligned using ClustalW [39]: various protein comparison matrix (e.g., Gonnet, Blosum and PAM) were tested and for each type of matrix, various combinations of the parameter values (*i.e*., the penalty for opening, closing, extending a gap and for the gap separation) were used. The alignments obtained with the Gonnet 250 matrix with gap opening and extension value of 1 and default value for the other parameters were chosen on the basis of both the scores calculated and "eye" inspection of the alignments. The full consensus sequence of each subset was determined with a consensus level > 60%. The 15 full consensus sequences were then aligned using the same parameters. Pairwise identity percentages were calculated with JalView Multiple Alignment Editor from ClustalW. The alignment of sequences of each subset and that of full consensus sequences related to this work have been deposited in the EMBL-Align Database and can be accessed through this database under # ALIGN_000563 and no. ALIGN_000564.

In order to compare the common structure to all GDH subunits called the central domain, the full consensus sequence of the smallest GDH subunit (subset I3, GDH not «EC-classified») was used as the template to extract from each other subset the consensus sub-sequence corresponding to this common central domain. The 15 consensus sub-sequences were themselves aligned using the same parameters as described above. The search for motifs and secondary structures was made with the ESPript program [40] using as the template the structure of bovine GDH3 complexed with NADPH and glutamate (Protein Data Bank # 1HWZ).

A theoretical 3D structure of GDH4 from *Chlorella sorokiniana *(subset *Ref*) was generated with the homology-modeling program ESyPred3D [41] using the structure of bovine GDH3 (PDB # 1HWZ) as the template. The modeling and the drawing of the putative structure of the active center of GDH4 from *Chlorella sorokiniana *were performed with the protein structure homology-modeling program DeepView (SwissPdb-Viewer v. 3.7) [42].

## Competing interests

The author(s) declare that they have no competing interests.

## Reviewers' comments

### Reviewer's report 1

Constantina Bakolitsa, Burnham Institute for Medical Research, CA 92121, USA

I find your revised version much improved. You have addressed my remarks adequately, with the exception of Figure 3 which I still think could benefit from a clearer representation.

Author's Response

*I do not agree. This figure is complex because it describes the co-existence of two NAD(P)H – binding sites and their interactions with various residues involved in the stabilization of the coenzyme*.

A couple of other points that might help further improve your manuscript.

1. You still need to check for spelling/grammatical typos.

Author's Response

*Language errors have been corrected*.

2. Your conclusion could benefit from having a few more sentences added summarizing your work prior to looking at future implications. Something perhaps like this: «The present study identifies a new coenzyme binding site in GDH4 with a potential regulatory role in GDH4 activity. The existence of such a site allows for the design of genetic engineering experiments that could potentially improve the efficiency of absorption and transportation of nitrogen. Other possible applications include enhancing the resistance of plants to environmental stresses such as dehydration, elevated CO2 levels and hypoxia. Further experiments will be required to address these issues.»

Author's Response

*The conclusion has been modified in order to take into account this important remark*.

### Reviewer's report 2

Martin Jambon, The Burnham Institute for Medical Research, CA 92037, USA

*Subject: *This article presents a computational analysis of the glutamate dehydrogenase (GDH). This enzyme comes in 3 forms, classified according to its coenzyme specificity (NAD mostly: EC 1.4.1.2 or NAD-GDH, here denoted GDH2; NAD or NADP: EC 1.4.1.3, denoted GDH3; NADP mostly: EC 1.4.1.4 or NADP-GDH, denoted GDH4).

The analysis is concerned by the role of GDH4 in plants, as it plays a role in ammonium assimilation and its importance with respect to the glutamine synthetase/glutamate synthase pathway is unclear. To date, there is no crystal structure of GDH4, and the only known gene in plants comes from *Chlorella sorokiniana*, and leads to two isoforms.

*Findings: *The author conducted a sequence analysis of the GDH family and classified them into several groups according to their size and coenzyme specificity. A representative from the GDH3 subset was carefully chosen to serve as a template for building a theoretical 3D model of GDH4 from *C. sorokiniana*.

Besides analyzing functional motifs that are know from other GDHs, the author proposes and discusses the presence of a putative second NADPH binding site, based on the similarity with an aldehyde dehydrogenase.

*Criticism: *This study appears to have been conducted carefully, and brings an interesting perspective toward understanding the role and the regulation of the NADP-GDH in plants. This certainly should be published. This study does not generate new experimental results but proposes models that would be useful for future experimentations. This is why it would be interesting to see diagrams for proposed models that would explain structure-function relationships. In particular, is the role of the putative second NADPH binding site to activate the enzyme ? It would be interesting to draw rough scenarios of which cellular contexts could cause the activity or inactivity of the enzyme, and how it is possible that the enzyme is used more for ammonium assimilation than the opposite reaction.

Author's Response

*The role of the putative second NADPH binding site is likely to activate the enzyme. The end of the discussion section has been re-written in that sense. It seems difficult, through a computational analysis, to draw such scenarios. I hope that this study can initiate further experimental approaches that will allow it. In particular, the first purification of GDH4 from plant, followed by its biochemical, enzymatic and cristallographic charaterization*.

English language and typos.

Author's Response

*Language errors have been corrected*.

### Reviewer's report 3

Sandor Pongor, International Centre for Genetic Engineering and Biotechnology, Italy

1. Generally speaking, computational analysis of protein families is always informative, in this respect the ms can be considered for publication, especially as part of a general review on the given protein family. I am not sure if a review supported with computational details is within the scope of Biology Direct. This work falls somewhat short of that aim, there is no systematic description of the pertinent literature, e.g.:

[a] Fontaine JX, Saladino F, Agrimonti C, Bedu M, Terce-Laforgue T, Tetu T, Hirel B, Restivo FM, Dubois F. Control of the synthesis and subcellular targeting of the two GDH genes products in leaves and stems of Nicotiana plumbaginifolia and Arabidopsis thaliana. Plant Cell Physiol. 2006 Mar;47(3):410–8. Epub 2006 Jan 17

[b] Masclaux-Daubresse C, Reisdorf-Cren M, Pageau K, Lelandais M, Grandjean O, Kronenberger J, Valadier MH, Feraud M, Jouglet T, Suzuki A. Glutamine synthetase-glutamate synthase pathway and glutamate dehydrogenase play distinct roles in the sink-source nitrogen cycle in tobacco. Plant Physiol. 2006 Feb;140(2):444–56. Epub 2006 Jan 11.

[c] Cruz C, Bio AF, Dominguez-Valdivia MD, Aparicio-Tejo PM, Lamsfus C, Martins-Loucao MA. How does glutamine synthetase activity determine plant tolerance to ammonium? Planta. 2006 Apr;223(5):1068–80. Epub 2005 Nov 16.

[d] Miflin BJ, Habash DZ. The role of glutamine synthetase and glutamate dehydrogenase in nitrogen assimilation and possibilities for improvement in the nitrogen utilization of crops.J Exp Bot. 2002 Apr;53(370):979–87. Review.

[e] Stitt M, Muller C, Matt P, Gibon Y, Carillo P, Morcuende R, Scheible WR, Krapp A. Steps towards an integrated view of nitrogen metabolism. J Exp Bot. 2002 Apr;53(370):959–70. Review.

[f] Suzuki A, Knaff DB. Glutamate synthase: structural, mechanistic and regulatory properties, and role in the amino acid metabolism. Photosynth Res. 2005;83(2):191–217. Review

Author's Response

*Four papers ([b], [c], [d] and [f]) suggested by Dr. Pongor were already added in the revised version (Ref. N° *[32,35,6]*and *[34]*, respectively). Concerning the two other ones (not added), some papers, maybe older but more original, were prefered*.

2. The work is about the structural features of GDH that can be predicted from computational analysis. It is not entirely clear to me what the main reason and the main goal of this analysis is. The author mentions, first in the title itself that a key role of GDH is suggested in this work. It is not apparent for me what this key role is, and how it can be related to the findings of this paper. One of the findings, the lack of the antenna region is not unique: non-mammalian GDH-s are generally knwon to lack the antenna regions.

Author's Response

*First, as mentioned above by Dr Pangor himself, a computational analysis of protein families is always informative. Secondly, the goal of this study was to link some structural features of GDH4 to the reaction catalysed by this isoform (the assimilation of ammonium into glutamate). Third, the main finding is the putative second NAD(P)H – binding site together with four residues involved in the stabilization of the coenzyme. The increase of both the expression and the activity of GDH observed in some ammonium conditions is likely the result of interactions between the two sites, allowing allosteric regulation of the enzymatic activity of GDH4 in the absence of antenna*.

3. The presentation of work is concise, the methodological details are put into the Appendix, which facilitates the reading of the work. The organization of the paper is not entirely clear. For instance, in the background there is a full summary of the conclusions. I do not see why this part belongs there. Some of the references are incomplete (publication year missing).

Author's Response

*The text has been largely reduced and the message has been concentrated (most of the redundancy was removed). A global organization chart summarizing the key structural features of the central domain has been added in Figure *1*. The alignment focalizes on the two coenzymes binding-sites. Four references have been added, mainly for argumentation of the controversy. I did not noticed any missing publication year in the revised version*.

### Reviewer's report 4

Frank Eisenhaber, Research Institute of Molecular Pathology (IMP), Vienna, Austria

The focus in this MS as described by the author is set on the role of GDHs in plants in the process of nitrogen assimilation. Targeting this goal with a sequence-analytic studies of various GDHs is problematic since it is known that these enzyme catalyze the reaction described on page 3 of this MS and the relative share of GDHs in the N-assimilation process is unlikely to be determined within the protein sequence of the GDHs themselves. Thus, this work will not contribute to this point. In this context, the reviewer wonders that the paper Glevarec et al. Planta (2004) 286–297 is not referred to.

Author's Response

*I agree with the remark concerning the paper of Glevarec et al. When it was published, it seemed that GDH4 has no role in ammonium assimilation. Then, a lot of work has been published suggesting that it may have a key role. This paper is now mentioned*.

The analysis of protein sequences of various subgroups of GDHs and the relationship of sequence patterns with function is another aspect of this MS; this question is more likely to be solved with the methods used in this work.

The collection of the sequence set that is the object of study is a critical point. It is the state of the art to collect the family by statistically rigorous similarity criteria applied on homologous sequence segments (in this case, apparently the central domain). For example, the BLAST/PSI-BLAST suite can be used:

Schaffer AA, Aravind L, Madden TL, Shavirin S, Spouge JL, Wolf YI, Koonin EV, Altschul SF. Improving the accuracy of PSI-BLAST protein database searches with composition-based statistics and other refinements. Nucleic Acids Res. 2001 Jul 15;29(14):2994–3005.

Altschul SF, Koonin EV. Iterated profile searches with PSI-BLAST – a tool for discovery in protein databases. Trends Biochem Sci. 1998 Nov;23(11):444–7.

Altschul SF, Madden TL, Schaffer AA, Zhang J, Zhang Z, Miller W, Lipman DJ. Gapped BLAST and PSI-BLAST: a new generation of protein database search programs. Nucleic Acids Res. 1997 Sep 1;25(17):3389–402.

It is unclear what kind of evidence supports the statements in the description lines, the similarity of the hydrophobic pattern and the conservation of critical functional residues are stronger arguments for structural and functional similarity. By ignoring non-annotated sequences, the author removes possibly important information about sequence variability and sequence knowledge about isoforms in some organisms.

As a next step, the family is subgrouped into clusters by sequence similarity criteria applied on the homologous segment. This is possible with programs such as CDhit, MCL or JACOP. Obvious cases can also be clustered manually. As distance criterion, the similarity determined with BLAST can be used.

Li W, Godzik A. cd-hit: a fast program for clustering and comparing large sets of protein or nucleotide sequences. Bioinformatics. 2006 May 26

Li W, Jaroszewski L, Godzik A. Sequence clustering strategies improve remote homology recognitions while reducing search times. Protein Eng. 2002 Aug;15(8):643–9

Sperisen P, Pagni M. JACOP: a simple and robust method for the automated classification of protein sequences with modular architecture. BMC Bioinformatics. 2005 Aug 31;6:216

Enright AJ, Van Dongen S, Ouzounis CA. An efficient algorithm for large-scale detection of protein families. Nucleic Acids Res. 2002 Apr 1;30(7):1575–84

The reviewer suggest that the subfamilies should resemble the GDH2-4 classification to some extent. Conservation of functional residues and, possibly, similarities in the sequence architectures within a subfamily (the sequence pieces outside the homologous domain) might support this clustering independently. Functional properties can possibly transferred within these subfamilies, e.g. the EC numbers.

Author's Response

*I have carefully read Dr Eisenhaber's remarks, as well as its book chapter "Prediction of Protein function", and I agree with these accute observations. However, I feel that they do not apply to my work. Dr Eisenhaber describes a very elegant strategy for a completely unknown amino acids (or nucleotide) sequence for which one wants to discover its function trough its structure*.

*In this work, the dataset is clearly defined : all sequences correspond to the same enzyme (GDH) and moreover for most of them, the structure is known and even the enzymatic specificity at the EC number level. The goal of my work was to identify structural features specific of the isoform GDH EC 1.4.1.4 from plants in order to demonstrate its putative key role in ammonium assimilation*.

*Concerning the removing of non-annotated sequences, I do not agree. Keeping in the data set the sequences annotated as «putative», «unknown protein », etc..., would have almost decreased the precision of the information*.

The conclusion about a second co-enzyme binding site is, at present, of speculative nature since a sequence pattern conservation detail and a 3D modeling study provide just plausibility.

Author's Response

*It seems true for all computational analysis*.

## Supplementary Material

Additional File 1 Appendix 1. Table containing the name of the organism, the EC number, the length in amino acids and the GenBank accession number, for each of the 116 non-redundant complete GDH sequences used in this study. Click here for file
