# Supplementary material for: A computational analysis of the three isoforms of glutamate dehydrogenase reveals structural features of the isoform EC 1.4.1.4 supporting a key role in ammonium assimilation by plants
Source: Biol Direct. 2006 Dec 15;1:38. doi: 10.1186/1745-6150-1-38 (PMC1716157; doi:10.1186/1745-6150-1-38)
Supplement: Additional File 1 — Appendix 1. Table containing the name of the organism, the EC number, the length in amino acids and the GenBank accession number, for each of the 116 non-redundant complete GDH sequences used in this study. [file 1745-6150-1-38-S1.pdf]

Appendix 1

**Characteristics of glutamate dehydrogenases used in this study. EC number, length and accession number of the 116 GDH sequences used in this work and names of the 83 organisms to which they belong.**				
subset	Organism	EC number	Amino acids	GenBank accession no.
**Ref**	*Chlorella sorokiniana* (*E*[a])	1.4.1.4	523	***CAA41635***
			523	***CAA41636***
**A**	*Asparagus officinalis (E)*	1.4.1.2	411	***CAA09478***
	Lycopersicon esculentum (E)		411	***AAL36888***
			412	***AAB39508***
	Nicotiana tabacum (E)		411	***CAD12373***
	Nicotiana plumbaginifolia (E)		411	***T16981***
			411	***CAB94837***
	Vitis vinifera (E)		411	***CAC18730***
	Zea mays (E)		411	***T04342***
			411	***T03294***
**B**	*Clostridium difficile (B)*	1.4.1.2	421	***S28829***
	Clostridium tetani (B)		421	***NP_781924***
	Fusobacterium nucleatum (B)		425	***EAA23508***
			439	***NP_603385***
	Haloferax mediterranei (A)		441	***CAC43018***
	Oceanobacillus iheyensis (B)		426	***BAC13766***
	Peptoniphilus asaccharolyticus (B)		421	***A38168***
**C**	*Brucella melitensis (B)*	1.4.1.2	1600	***AAL51413***
**D**	*Vitis vinifera (E)*	1.4.1.3	411	***S54797***
**E**	*Pyrococcus furiosus (A)*	1.4.1.3	420	***NP_579331***
	Sulfolobus solfataricus (A)		420	***NP_343440***
			434	***NP_343336***
			420	***NP_343309***
			419	***NP_342894***
	Thermotoga maritima (B)		416	***CAA71058***
**F**	*Chaenocephalus aceratus (B)*	1.4.1.3	504	***P82264***
	Drosophila melanogaster (E)		549	***S42919***
	Gallus gallus (E)		503	***P00368***
	Homo sapiens (E)		558	***CAA46995***
	Mus musculus (E)		558	***NP_032159***
	Salmo salar (E)		544	***CAD58716***
			539	***CAD58714***
**G**	*Agaricus bisporus (E)*	1.4.1.4	457	***S63608***
	Bacillus halodurans (B)		458	***NP_242967***
	Bacillus licheniformis (B)		460	***AAC69873***
	Corynebacterium glutamicum (B)		447	***NP_601279***
	Escherichia coli (B)		447	***NP_754056***
			447	***DEECEN***
	Giardia intestinalis (E)		449	***A42489***
	Haemophilus influenzae (B)		449	***A64053***
	Laccaria bicolor (E)		450	***P54388***
	Penicillium chrysogenum (E)		461	***AAF00006***
	Salmonella typhimurium (B)		447	***AAL20224***
	Streptococcus pneumoniae (B)		448	***NP_358774***

**G**	*Trypanosoma cruzi (E)*	1.4.1.4	446	***AAC14692***
			446	***AAC14691***
	Tuber borchii (E)		457	***AAG28788***
**H**	*Arabidopsis thaliana (E)*	NC	411	***NP_196361***
	Brassica napus (E)		411	***BAB62170***
	Nicotiana plumbaginifolia (E)		411	***T16982***
	Oryza sativa (E)		411	***AAO37984***
	Ulva pertusa (E)		421	***BAB62312***
			447	***BAB62311***
**I1**	*Bacillus halodurans (B)*	NC	430	***NP_243584***
	Bacillus halodurans (B)		420	***NP_244810***
	Bacillus halodurans (B)		421	***NP_242488***
	Chlorobium tepidum (B)		418	***NP_662898***
	Pyrococcus abyssi (A)		420	***NP_126260***
	Pyrococcus endeavori (A)		420	***AAA64795***
			420	***A47410***
	Staphylococcus aureus (B)		414	***NP_374080***
	Staphylococcus epidermidis (B)		414	***NP_764209***
	Synechocystis sp. PCC6803 (B)		428	***NP_442685***
	Thermococcus waiotapuensis (A)		419	***AAK53112***
	Thermoplasma volcanium (A)		416	***BAB59913***
			435	***BAB59912***
**I2**	*Bacteroides thetaiotaomicron (B)*	NC	444	***NP_810883***
	Bifidobacterium longum (B)		448	***NP_695816***
	Clostridium acetobutylicum (B)		443	***NP_347373***
	Corynebacterium efficiens (B)		466	***BAB86838***
	Deinococcus radiodurans (B)		424	***E75362***
	Entodinium caudatum (E)		438	***AAF15393***
	Escherichia coli (B)		447	***NP_288194***
			447	***AAA23868***
	Klebsiella aerogenes (B)		447	***AAK17986***
	Plasmodium falciparum (E)		470	***NP_702052***
			442	***AAG12331***
			442	***AAD11789***
	Pseudomonas aeruginosa (B)		445	***NP_253278***
	Ruminococcus flavefaciens (B)		455	***AAN15204***
	Salmonella enterica (B)		447	***NP_804986***
	Streptococcus agalactiae (B)		449	***NP_688333***
	Streptococcus pneumoniae (B)		448	***NP_345769***
	Streptococcus suis (A)		448	***AAK00759***
**I3**	*Bacteroides fragilis (B)*	NC	445	***AAC26399***
	Bacteroides thetaiotaomicron (B)		445	***NP_810886***
	Botryotinia fuckeliana (E)		450	***AAC95390***
	Debaryomyces hansenii (E)		418	***AAF98446***
	Gibberella fujikuroi (E)		451	***CAC45043***
	Helicobacter pylori (B)		448	***D64567***
			448	***F71862***
	Neurospora crassa (E)		454	***EAA32325***
			454	***1003201A***
	Neurospora intermedia (E)		454	***AAG01158***
	Neurospora sitophila (E)		454	***AAG01159***
			454	***NP_015020***
	Saccharomyces cerevisiae (E)		453	***1111238A***
			454	***AAB03898***

**J**	*Bos taurus (E)*	NC	520	***AAN15276***
	Homo sapiens (E)		558	***CAA30521***
	Oncorhynchus mykiss (E)		539	***AAM73777***
			544	***AAM73775***
	Rattus norvegicus (E)		558	***CAA32202***
**K**	*Agaricus bisporus (E)*	NC	1029	***CAB40797***
	Gibberella fujikuroi (E)		1059	***CAC27837***
	Neurospora crassa (E)		1047	***AAB28355***
	Saccharomyces cerevisiae (E)		1092	***NP_010066***
	Schizosaccharomyces pombe (E)		1106	***T40931***
**L**	*Caulobacter crescentus (B)*	NC	1607	***NP_418907***
	Mycobacterium leprae (B)		1622	***NP_301902***
	Mycobacterium tuberculosis (B)		1624	***NP_337039***
	Pseudomonas aeruginosa (B)		1620	***AAG53963***
	Streptomyces avermitilis (B)		1645	***BAC72787***
	Streptomyces clavuligerus (B)		1651	***AAG40620***
	Vibrio cholerae (B)		1613	***NP_231133***

[a]A, Archaea - B, Bacteria - E, Eukaryota.
